# Supplementary material for: A Novel Mechanically Robust and Biodegradable Egg White Hydrogel Membrane by Combined Unidirectional Nanopore Dehydration and Annealing
Source: Int J Mol Sci. 2023 Aug 10;24(16):12661. doi: 10.3390/ijms241612661 (PMC10454319; doi:10.3390/ijms241612661)

Figure S1. The photos of UND-based EWGs from five eggs, (**a**) ostrich, (**b**) goose, (**c**) duck, (**d**) chicken, and (**e**) quail eggs.

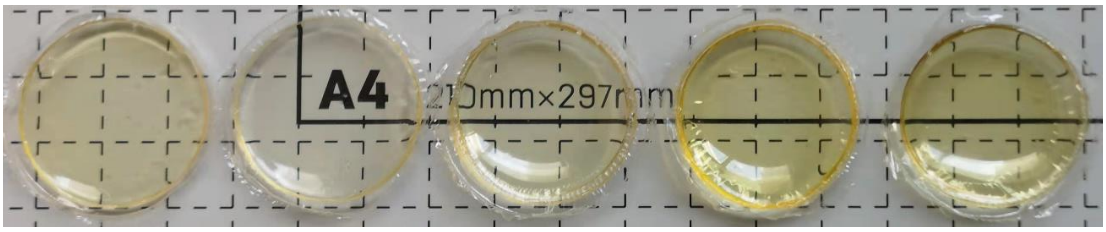

Supplement: Supplementary file 1 [file ijms-24-12661-s001.zip › Figure S1.pdf]
